# A Prospective Comparative Study of Shouldice Inguinal Hernia Repair and Total Transabdominal Preperitoneal (TAPP) Laparoscopic Inguinal Hernia Repair: A Single-Center Experience

**DOI:** 10.7759/cureus.93679

**Published:** 2025-10-01

**Authors:** Chintankumar Patel, Dinesh Prasad, Vipul D Yagnik, Prema R Choudhary

**Affiliations:** 1 Surgery, Surat Municipal Institute of Medical Education and Research, Surat, IND; 2 General Surgery, Surat Municipal Institute of Medical Education and Research, Surat, IND; 3 Surgery, Banas Medical College and Research Institute, Palanpur, IND; 4 Physiology, Banas Medical College and Research Institute, Palanpur, IND

**Keywords:** inguinal hernia, inguinodynia, shouldice repair, testicular atrophy, transabdominal preperitoneal (tapp)

## Abstract

Background: More than 20 million hernias are repaired annually. This study aimed to compare two techniques, Shouldice hernia repair and transabdominal preperitoneal (TAPP) laparoscopic hernia repair, in terms of various measures, such as recurrence rates, intraoperative and postoperative complications, and length of postoperative stay.

Methods: A single-center, prospective, comparative study was conducted among patients admitted to a tertiary care hospital with a diagnosis of primary inguinal hernia (direct or indirect). A total of 125 patients underwent surgery. Among these patients, 53 had bilateral inguinal hernias, and 72 had unilateral inguinal hernias. The patients were alternatively allocated (based on their outpatient department visits) to either TAPP laparoscopic hernia repair or Shouldice hernia repair. All patients were followed up both in the short term (on postoperative days 1, 3, and 15) and in the long term (at 6 and 12 months) on an outpatient basis. Follow-up included a clinical examination and ultrasonography to assess recurrence and complications, such as postoperative pain, seroma formation, testicular atrophy, inguinodynia, foreign body sensation, and recurrence.

Results: The mean operative durations of the Shouldice and laparoscopic TAPP groups were 82.7 ± 12.9 minutes and 107.6 ± 13.8 minutes, respectively, indicating that significantly less time was needed for Shouldice repair (p = 0.001). Postoperative pain was measured on days 1, 3, and 15, and visual analog scale scores were compared. Comparing the two procedures for pain on days 1, 3, and 15, there was less pain associated with the Shouldice repair than with the TAPP repair. However, this difference was not statistically significant (p = 0.2199). Seroma formation and testicular atrophy were significantly greater in the TAPP group than in the Shouldice group (p = 0.047 and p = 0.0194, respectively). Inguinodynia was significantly greater in the laparoscopic group than in the Shouldice repair group (p = 0.029). At the 12-month follow-up, there was one case of recurrence after Shouldice hernia repair (1.1%) and two cases of recurrence after TAPP laparoscopic hernia repair (2.2%). This difference was not statistically significant (p > 0.05).

Conclusion: The operative time was significantly shorter for Shouldice than for laparoscopic TAPP. Postoperative pain and recurrence rates were comparable in both groups, whereas the seroma and testicular atrophy rates were higher in the laparoscopic TAPP group.

## Introduction

The lifetime prevalence of groin hernias is estimated to be 27%-43% in males and 3%-6% in females [1], with 1.6 million diagnosed annually and 500,000 undergoing operative repair in the United States [2]. Transabdominal preperitoneal (TAPP) laparoscopic prosthetic repair using polypropylene mesh is a popular minimally invasive laparoscopic method. However, it is not a tissue-based repair method; mesh displacement is the leading cause of recurrence, and mesh-related complications (risk of infection, recurrence, chronic pain, testicular atrophy and infertility, and foreign body sensations) are the leading causes of morbidity. The mesh does not provide a mobile and physiologically dynamic posterior wall [3]. Recent updates to the International HerniaSurge guidelines for groin hernia management recommend TAPP as the preferred technique for inguinal hernia repair, while preserving the Shouldice technique for selected groups of patients [4].

Tissue repair methods, such as modified Bassini, iliotibial tract repair, Desarda repair, nylon darn, Halsted-Tanner repair, and McVay repair, require good surgical experience or tension repair, as they can lead to relapses. Relapses vary from surgeon to surgeon and from center to center because of the complexity of the procedures. During World War II, in the 1940s, the Canadian Earle Shouldice developed the Shouldice repair [5]. As the abdominal muscles contract, the aponeurosis strip provides more physiological support to the posterior wall. Theoretically, this procedure is closer to ideal hernia repair.

Laparoscopic inguinal hernia repair, particularly the TAPP approach, is well recognized to have a steep learning curve, with most studies indicating that proficiency is achieved after approximately 30-50 supervised cases, while mastery requires considerably higher case volumes [6]. This reflects the technical demands of laparoscopic anatomy, camera navigation, intracorporeal dissection, and mesh placement. In contrast, the Shouldice repair, although technically demanding, demonstrates a different learning trajectory. Analyses from the Shouldice Hospital report that operative times plateau after 78-88 procedures, with full surgical proficiency generally achieved after around 1,000 repairs [7]. Thus, while TAPP requires fewer repetitions to reach competency, it is technically more complex and dependent on advanced equipment, whereas Shouldice repair demands extensive repetition for consistency but remains more accessible in open, resource-limited settings.

A few complications that are exclusively limited to laparoscopic TAPP, not open tissue repair, include small bowel obstructions and trocar site hernias [8,9]. Bleeding can occur with both techniques, but it is more troublesome in TAPP laparoscopic repair. The spiral tack used to repair the peritoneum in TAPP has been found to erode adjacent viscera [9].

Shouldice repair remains a durable and cost-effective alternative to mesh-based techniques, offering comparable outcomes in selected patients. However, evidence from non-specialized centers is limited, underscoring the need for prospective studies to better define its role in broader clinical practice. Evidence on recurrence, chronic pain, testicular atrophy, and foreign body sensation remains sparse. This prospective comparative study was therefore designed with clearly defined objectives: the primary outcomes were recurrence rate and long-term postoperative complications (inguinodynia, testicular atrophy, and foreign body sensation). The secondary outcomes included intraoperative variables, early postoperative pain, seroma formation, and operative duration.

## Materials and methods

Method

This single-center, prospective, comparative study was conducted at a tertiary care hospital in Surat between December 5, 2021 and December 5, 2022, following approval from the institutional ethics committee. Patients older than 18 years with primary inguinal hernias (direct or indirect) were eligible. Exclusion criteria included age <18 years, unwillingness to undergo Shouldice or TAPP laparoscopic repair, complicated or congenital hernias, and pregnancy. All patients first underwent a detailed history and clinical examination, and hernias were classified according to the Nyhus classification [10]. All procedures and evaluations were performed by experienced consultant surgeons, each of whom had independently performed at least 100 Shouldice repairs and 50 laparoscopic TAPP procedures before this study. After confirming eligibility, the study purpose and treatment options were explained, and written informed consent was obtained. Patients were then allocated alternately, based on the sequence of their outpatient visits, to either a Shouldice repair or a laparoscopic TAPP procedure. The study adhered to the principles of the Declaration of Helsinki. The distribution of unilateral and bilateral cases between the two groups was compared to account for potential confounding, and subgroup analyses were performed to assess operative duration separately for unilateral and bilateral repairs. Contact details were recorded to ensure follow-up. All patients were evaluated for operative findings and postoperative complications, with follow-up visits scheduled on postoperative days 1, 3, and 15, and subsequently at 6 and 12 months. These visits included clinical examination and ultrasonography to detect recurrence and complications. Definitions used included inguinodynia (pain persisting beyond three months, assessed by visual analog scale (VAS)), foreign body sensation (localized discomfort assessed subjectively), seroma (fluid collection detected clinically and confirmed by ultrasonography), and testicular atrophy (>20% reduction in testicular volume on ultrasound).

Procedure

All patients undergoing laparoscopic TAPP had the procedure performed under general anesthesia, using a 15 × 12 cm microporous polypropylene mesh (Prolene Mesh, Ethicon, Johnson & Johnson Pvt. Ltd., Somerville, New Jersey, USA), which was secured with tacks for fixation. Shouldice repair was performed under spinal anesthesia, in which the posterior wall was reinforced by double breasting the transversalis fascia and strengthening the inguinal ligament with the conjoint tendon using 2-0 polypropylene sutures (Prolene, Ethicon, Johnson & Johnson Pvt. Ltd., Somerville, New Jersey, USA). All patients received intramuscular diclofenac sodium 75 mg every 12 hours for 48 hours, followed by oral paracetamol 650 mg twice daily for 3 days. A single preoperative intravenous dose of ceftriaxone 1 g was also administered, and no opioids were used. The operative time was measured from the time the skin incision was made until the final suture was used for skin closure.

The patients were meticulously followed for both short-term and long-term outcomes. Short-term assessments included postoperative pain, measured on days 1, 3, and 15, and seroma formation. Long-term follow-up focused on the evaluation of chronic pain (inguinodynia at 6 and 12 months), testicular atrophy, foreign body sensation, and recurrence. Postoperatively, the wound was inspected, and dressings were applied 72 hours after surgery and subsequently every 48 hours. Swelling at the wound site, discharge, discoloration, and scrotal swelling were noted. One or two sutures were removed, as needed, in cases of infection or seroma. Ultrasonography of the inguinoscrotal region was performed in patients with postoperative swelling. Hematoma or seroma was considered in patients with anechoic collection, with or without internal echoes. The patient was encouraged to ambulate as soon as possible after the effects of anesthesia had worn off. Patients were discharged only after fulfilling all criteria: absence of wound infection, ability to ambulate, tolerance of oral intake, and subjective comfort with discharge. While formal follow-up assessments in the study were performed on postoperative days 1, 3, and 15, suture removal between the 7th and 10th postoperative days was carried out as a standard surgical practice after careful inspection of the stitch line, rather than as part of the study protocol.

Sample size calculation

The study considered multiple primary endpoints, including the hernia recurrence rate, chronic postoperative pain (inguinodynia), and testicular atrophy, all of which were assessed at the 12-month follow-up. The primary sample size calculation was initially based on the recurrence rate, which is a critical long-term outcome of hernia surgeries. To detect a clinically significant difference of approximately 5% in hernia recurrence rates between Shouldice repair (~2%) and laparoscopic TAPP repair (~7%), with a power of 80% (β = 0.20) and an alpha error (two-sided) of 0.05, a minimum of 55 patients per group was needed.

However, recognizing the clinical importance of other outcomes, such as chronic postoperative pain and testicular atrophy, the calculated sample size was considered adequate, as it would simultaneously provide sufficient statistical power (>80%) to detect clinically relevant differences in these outcomes, assuming expected differences of at least 10%-15% based on previous studies.

After accounting for an anticipated dropout rate of approximately 10%, a total of 125 patients were enrolled, resulting in 178 hernia repairs (89 in each group), to ensure adequate power for evaluating the primary endpoints.

Statistical methods

All collected data were entered into a computerized database and analyzed using the Statistical Package for the Social Sciences (SPSS) version 26.0 (IBM Corp., Armonk, NY, USA). Descriptive statistics, including means with standard deviations for normally distributed continuous variables and medians with interquartile ranges for skewed distributions, were calculated to summarize patient demographics and baseline characteristics.

Comparisons between the laparoscopic TAPP and Shouldice repair groups were performed using independent Student’s t tests for continuous variables with normal distribution, and the Mann‒Whitney U test was applied for non-normally distributed continuous data. For categorical variables, such as postoperative complications and recurrence rates, the chi-square test was used. In cases where the expected cell frequencies were less than five, Fisher’s exact test was applied to ensure the validity of the results.

Normality of distribution was assessed using the Shapiro-Wilk test and by examining histograms and Q-Q plots. Homogeneity of variances was tested with Levene’s test before applying parametric tests. Missing data, if any, were handled using a complete-case analysis approach (listwise deletion).

All tests were two-tailed, and a p-value <0.05 was considered statistically significant. Results were presented as tables and figures where appropriate to enhance clarity.

## Results

A total of 125 patients underwent surgery; 53 presented with bilateral inguinal hernias and 72 with unilateral hernias, resulting in 178 hernia repairs overall. Of these, 47 direct hernias were treated with Shouldice repair and 54 with laparoscopic TAPP, while 42 indirect hernias were treated with Shouldice repair and 35 with laparoscopic TAPP.

In our study, the mean age was 43.8 ± 12.6 years in the Shouldice group and 46.1 ± 15.1 years in the TAPP group. The mean duration of inguinal or inguinoscrotal swelling was 18.3 ± 10.1 months in the Shouldice repair group and 18.4 ± 9.9 months in the laparoscopic TAPP group.

The distribution of unilateral and bilateral hernia repairs was similar between the two groups (Shouldice: 41 unilateral, 24 bilateral; TAPP: 31 unilateral, 28 bilateral; p = 0.315). This difference was not statistically significant (p = 0.315). When operative time was analyzed separately by laterality, the difference between techniques persisted. For unilateral repairs, the mean operative duration was 82.44 ± 11.79 minutes in the Shouldice group compared with 106.77 ± 12.22 minutes in the TAPP group (p < 0.001). For bilateral repairs, the mean duration was 82.92 ± 13.83 minutes in the Shouldice group compared with 108.10 ± 14.68 minutes in the TAPP group (p < 0.001) (Table 1).

**Table 1:** Demographic and baseline characteristics of patients undergoing Shouldice repair and laparoscopic TAPP Values are expressed as mean ± standard deviation for continuous variables and as number (percentage) for categorical variables. *Independent t-test was used for continuous variables with normal distribution; Mann–Whitney U test was used for non-normally distributed continuous variables (duration of swelling and visual analog scale scores). †Chi-square test or Fisher’s exact test was applied for categorical variables. A p-value <0.05 was considered statistically significant. TAPP, Total transabdominal preperitoneal

Parameter	Shouldice repair (n=89)	Laparoscopic TAPP (n=89)	p-value
Mean age (years)	43.8 ± 12.6	46.1 ± 15.1	0.274*
Age groups, n (%)			0.090^†^
<40 years	33 (37.1%)	28 (31.5%)	
40–59 years	43 (48.3%)	36 (40.4%)	
≥60 years	13 (14.6%)	25 (28.1%)	
Gender, n (%)			1.000^†^
Male	87 (97.8%)	88 (98.9%)	
Female	2 (2.2%)	1 (1.1%)	
Mean duration of swelling (months)	18.3 ± 10.1	18.4 ± 9.9	–
Laterality, n (%)			0.315^†^
Unilateral	41 (63.1%)	31 (52.6%)	
Bilateral	24 (36.9%)	28 (47.4%)	
Operative duration (minutes)	82.7 ± 12.9	107.6 ± 13.8	0.001*
– Unilateral repairs	82.44 ± 11.79	106.77 ± 12.22	<0.001*
– Bilateral repairs	82.92 ± 13.83	108.10 ± 14.68	<0.001*

Comparison of postoperative complications between the two groups

**Figure 1:**
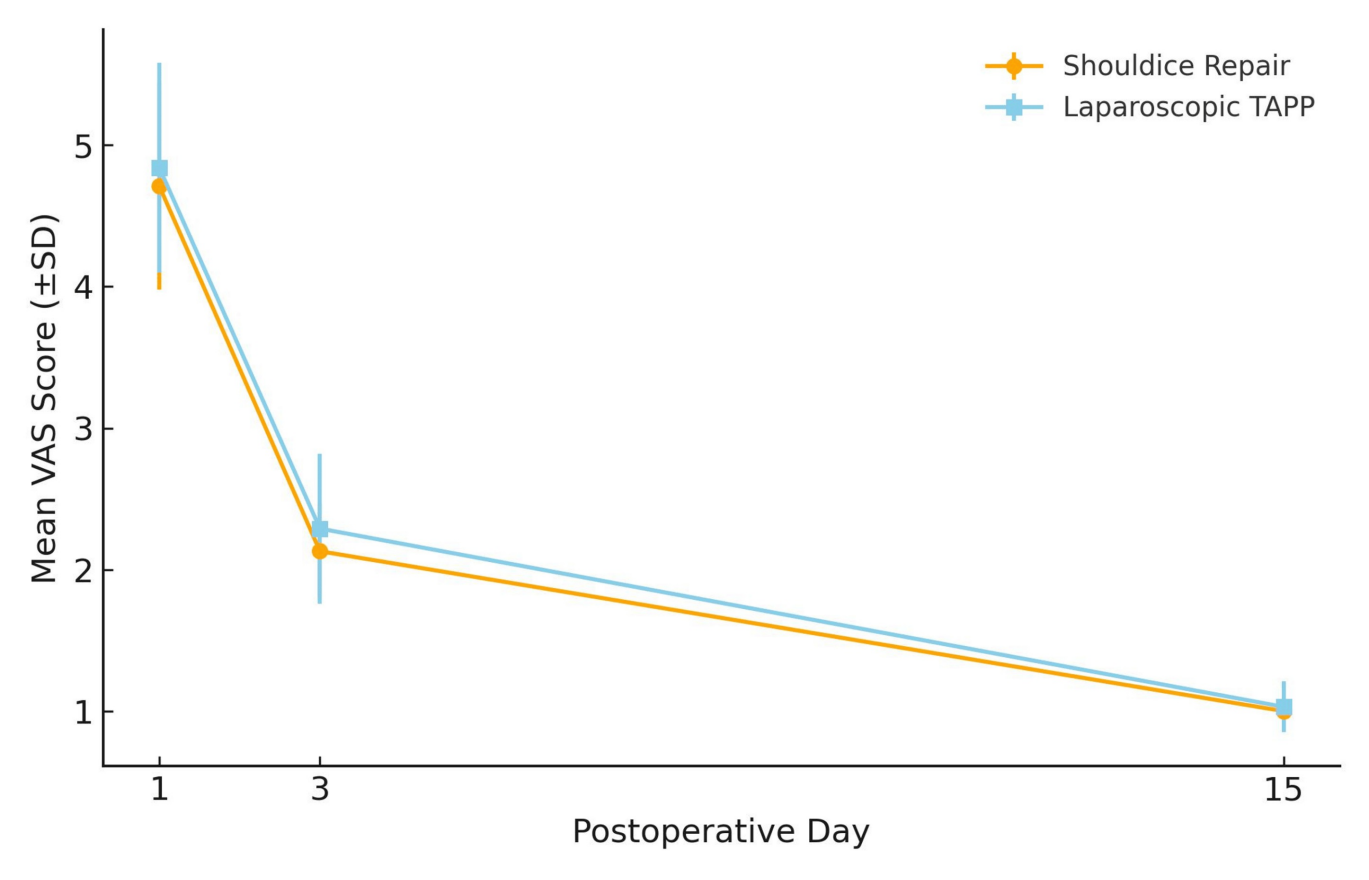
Comparison of postoperative pain between the two groups Line graph comparing postoperative pain scores (VAS) [11] between Shouldice repair and laparoscopic TAPP repair. Patients undergoing Shouldice repair reported consistently lower pain scores than those undergoing TAPP repair (p = 0.2199). SD, Standard deviation; TAPP, Transabdominal preperitoneal; VAS, Visual analog scale

Postoperative pain was measured on days 1, 3, and 15, and VAS scores [11] were compared, as shown in Figure 1. In the Shouldice repair group, mean VAS scores were 4.71 ± 0.73 on day 1, 2.13 ± 0.34 on day 3, and 1.00 ± 0.00 on day 15. In the laparoscopic TAPP group, the corresponding scores were 4.84 ± 0.74, 2.29 ± 0.53, and 1.03 ± 0.18. Although patients undergoing Shouldice repair reported consistently lower pain scores, the difference was not statistically significant (p = 0.2199) (Figure 1). The p-value was calculated using the Mann-Whitney U test, given the ordinal nature of the VAS scores.

When the two groups were compared, the incidence of seroma formation on days 1, 3, and 15 was significantly greater in five patients in the laparoscopic TAPP hernia group than in one patient in the Shouldice hernia group (p = 0.047) (Table 2).

**Table 2:** Comparison of the incidence of seroma formation between Shouldice repair and laparoscopic TAPP Values are expressed as the number of patients. *Statistical comparison was performed using Fisher’s exact test at each follow-up interval; only day 3 showed a statistically significant difference (p = 0.0474).

Follow-up day	Shouldice repair (n=89)	Laparoscopic TAPP (n=89)	p-value
Day 1	1	1	-
Day 3	0	2	0.0474*
Day 15	0	2	-
Total	1	5	-

At the six-month follow-up, testicular atrophy was observed in five patients undergoing laparoscopic TAPP hernia repair, but not in the Shouldice group. At the 12-month follow-up, testicular atrophy was observed in six patients in the laparoscopic TAPP hernia repair group and in only one patient in the Shouldice hernia repair group. This difference was statistically significant (p = 0.0194) (Table 3).

**Table 3:** Testicular atrophy at follow-up Values are expressed as the number of patients. *Chi-square test or Fisher’s exact test was applied depending on expected cell counts. A p-value <0.05 was considered statistically significant.

Follow-up	Shouldice repair (n=89)	Laparoscopic TAPP (n=89)	p-value
6 months	0	5	-
12 months	1	6	0.0194*

**Figure 2:**
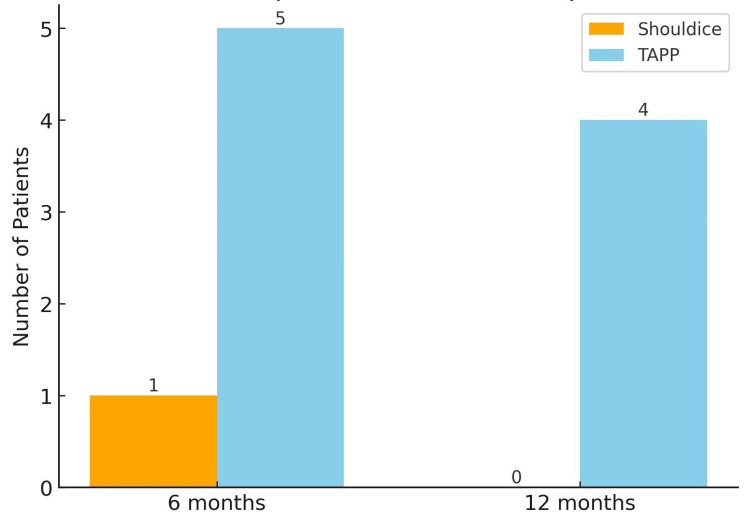
Inguinodynia at follow-up Bar chart showing the number of patients reporting inguinodynia (VAS ≥3) at 6 and 12 months after hernia repair. Inguinodynia was more frequent in the TAPP group than in the Shouldice group (p = 0.029). TAPP, Transabdominal preperitoneal; VAS, Visual analog scale

At the six-month follow-up, inguinodynia was assessed using the VAS [11]. A VAS score of 3 or higher is often considered the threshold for a positive assessment, indicating moderate-to-severe pain that impacts daily activities. It was observed in five patients in the laparoscopic TAPP hernia repair group and in one patient in the Shouldice hernia repair group. At the 12-month follow-up, inguinodynia was observed in only four patients in the laparoscopic TAPP hernia repair group and in none of the patients in the Shouldice hernia repair group, and the difference was statistically significant (p = 0.029) (Figure 2).

**Figure 3:**
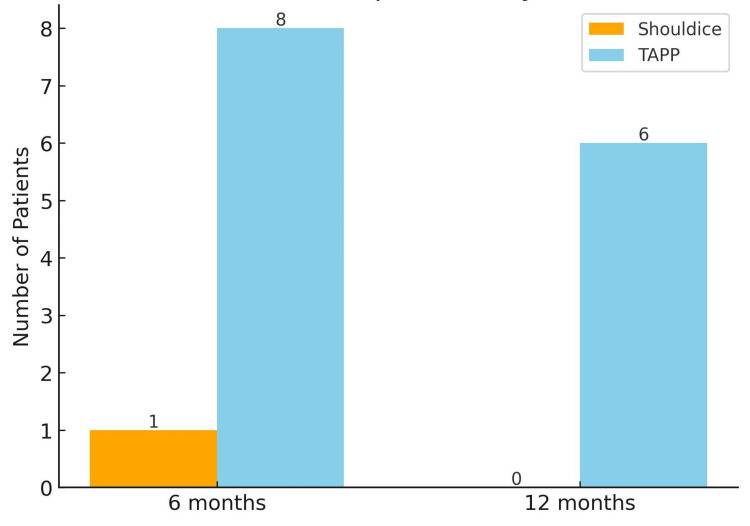
Foreign body sensation at follow-up Bar chart illustrating the number of patients experiencing foreign body sensation at 6 and 12 months postoperatively. The sensation was significantly higher among patients treated with TAPP repair compared with those treated with Shouldice repair (p = 0.0286 at 12 months). TAPP, Transabdominal preperitoneal

At the six-month follow-up, foreign body sensation was reported in 8/89 patients in the laparoscopic TAPP group (9.0%) compared with 1/89 in the Shouldice group (1.1%), a statistically significant difference (p = 0.0344). At 12 months, foreign body sensation persisted in 6/89 patients in the TAPP group (6.7%) and in 0/89 patients in the Shouldice group (0%), a statistically significant difference (p = 0.0286) (Figure 3). 

**Figure 4:**
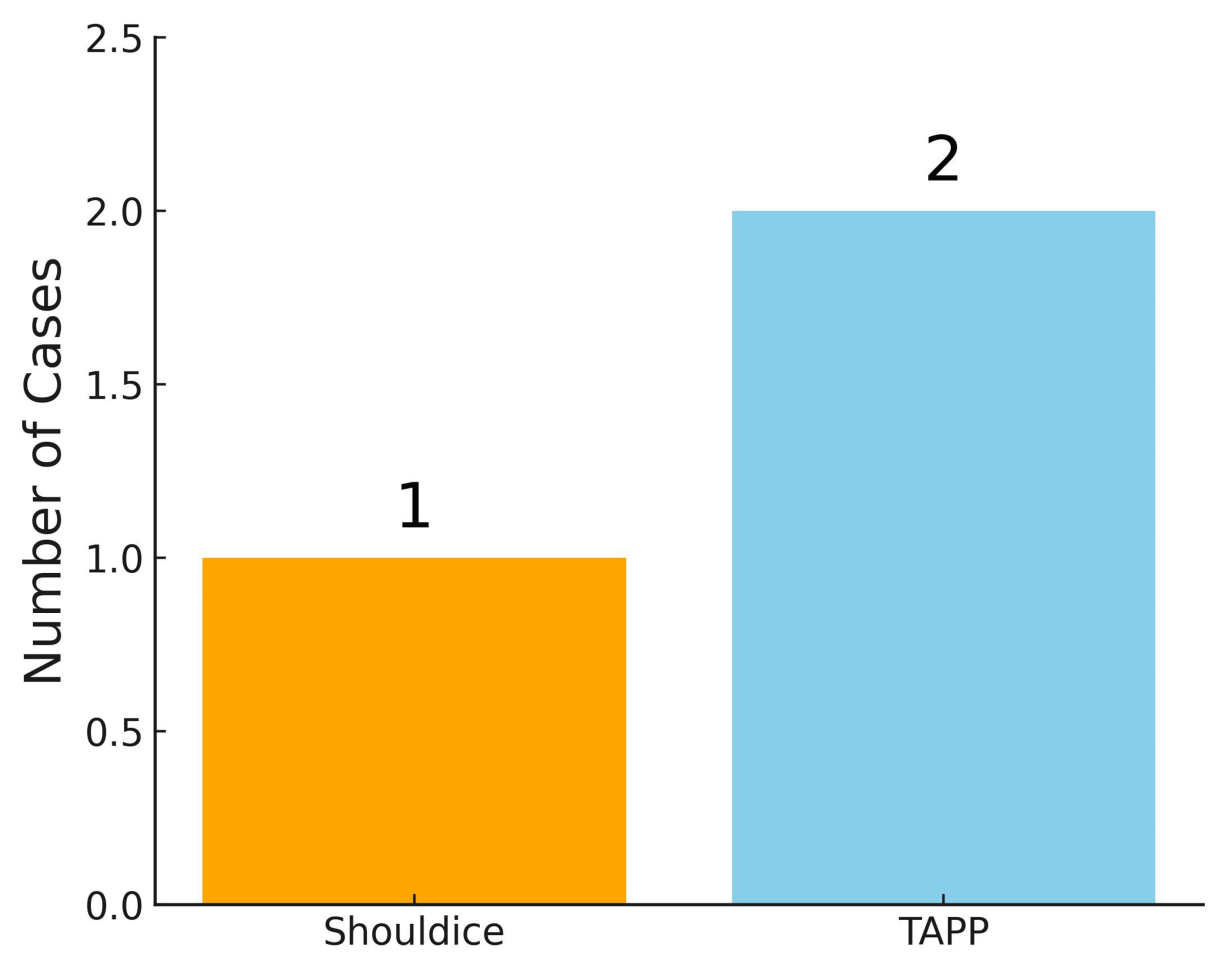
Recurrence at 12 months Bar chart demonstrating recurrence rates at 12 months after surgery. Recurrence was observed in both groups, with slightly higher cases in the TAPP group compared with the Shouldice group (p > 0.05).

At the 12-month follow-up, there was one case of recurrence after Shouldice hernia repair (1.1%) and two cases of recurrence after laparoscopic TAPP (2.2%). This difference was not statistically significant (p > 0.05) (Figure 4).

## Discussion

Despite the widespread use of meshes, pure tissue repair has been used in modern hernial surgery. Mesh has been associated with various complications, including infection, migration, rejection, foreign body sensation, and nerve entrapment. Some patients may be allergic to mesh, and in such cases, tissue-based repairs remain a valuable option, particularly in resource-limited settings where cost is a significant concern. Among the various pure tissue repairs, the Shouldice technique is associated with the lowest recurrence rates, reported as low as 2.88% after 18 years of follow-up, and moderate-to-severe pain in only 1.8% of cases after three years [12]. A recent study at Shouldice Hospital demonstrated a very low surgical site infection rate (1.96%) and a recurrence rate of 1.6% [13]. Furthermore, two high-quality, extensive studies have shown that the outcomes of Shouldice repair are comparable to those of mesh-based repairs such as TAPP, totally extraperitoneal (TEP), or Lichtenstein repair at one year of follow-up [14,15]. The cost-effectiveness of Shouldice repair further reinforces its practicality in resource-limited environments.

Despite its effectiveness, Shouldice repair has not been widely accepted as a universal technique for hernia repair due to its high recurrence rate in non-specialized centers [16]. A meta-analysis performed by Pompeu et al. also revealed a higher recurrence rate with Shouldice repair than with mesh repair [17]. This emphasizes the need for caution regarding the widespread use of the Shouldice repair. Mesh repair is associated with mesh-related complications, including seroma, infection, displacement, and increased costs. In addition to the complications mentioned above, mesh has been found to alter testicular and sexual functions [18]. Laparoscopic surgery has a better outcome in terms of preserving testicular and sexual function [18]. In our study, the mean duration of inguinal or inguinoscrotal swelling was 18.3 ± 10.1 months in the Shouldice repair group and 18.4 ± 9.9 months in the laparoscopic TAPP group. The operative time was significantly shorter in the Shouldice group than in the TAPP group (82.7 ± 12.9 minutes vs. 107.6 ± 13.8 minutes, respectively); however, there was no statistically significant difference in the pain score. In contrast, seroma formation and testicular atrophy were more prevalent in the TAPP group than in the Shouldice group. Inguinodynia was also more common in the laparoscopic group than in the Shouldice group. At the 12-month follow-up, there was one recurrence after Shouldice repair (1.1%) and two recurrences after TAPP (2.2%). These differences were not significant (p > 0.05).

A 2018 Cochrane Review by Lockhart et al. analyzed 25 randomized controlled trials involving 6,293 participants to compare mesh and non-mesh repair techniques for inguinal and femoral hernias. Mesh repair was associated with a significantly lower risk of hernia recurrence (risk ratio, 0.46; 95% confidence interval, 0.26-0.80) and fewer neurovascular or visceral injuries. However, mesh use showed slightly higher rates of seroma formation and wound swelling. Other complications, such as infection, hematoma, and testicular injury, showed no significant differences. Operative time, hospital stay, and return to regular activity varied across studies, and conclusions for these outcomes remain uncertain. The review concluded that both techniques are effective, with mesh repair preferred in many settings due to its lower recurrence rates [19].

In another study, 365 patients with primary unilateral inguinal hernias were randomized to one of the following five procedures: Shouldice repair, Bassini operation, Lichtenstein repair, laparoscopic transabdominal extraperitoneal hernioplasty (TEP), or TAPP. Patients were followed up at 12, 24, and 36 months to assess the cumulative recurrence rate and the rates of intraoperative, perioperative, and long-term complications [20]. No statistically significant differences were observed in the rates of intraoperative or perioperative and long-term complications among the five groups [20]. This highlights the variety of options available, each with comparable outcomes. One study randomly assigned 151 patients to the Shouldice, Bassini, or TAPP groups. Patients with Shouldice repairs had significantly higher pain scores on the VAS (p = 0.048) and on the McGill pain questionnaire on the first postoperative day (p = 0.046) than those in other groups [21]. Apart from the significantly lower score of postoperative bodily pain in the Shouldice group (p = 0.039), no significant differences in quality of life were apparent among the three methods [18]. An extensive database comparing Shouldice with TAPP or TEP revealed that the recurrence rate was comparable in selected patients [15]. Our study contributes further by offering a prospective, comparative analysis with 12-month follow-up that evaluates both early and long-term outcomes while accounting for confounders such as unilateral versus bilateral repairs.

These findings contrast with the observations from our study, where foreign body sensation, inguinodynia, and testicular atrophy at the 12-month follow-up were considerably lower in the Shouldice repair group than in the laparoscopic TAPP group. The higher incidence of inguinodynia in the TAPP group in our study may be attributed to mesh-related nerve irritation, fibrosis, and fixation techniques, such as tackers. Differences from the findings of Köckerling et al. are likely due to variations in patient populations, mesh types, fixation methods, and the definitions or assessment tools used for chronic pain across studies [15]. The tackers used to fix the mesh could also be a reason for the higher inguinodynia rate. The placement of a mesh in the preperitoneal space during TAPP may exert pressure on the spermatic cord or its vascular structures, and microscopic trauma that may not be immediately evident can lead to delayed ischemia and atrophy.

For primary unilateral inguinal hernia, contemporary guidance synthesizing randomized and real-world evidence indicates that laparoscopic-endoscopic repair (TEP/TAPP) provides faster recovery and lower rates of acute and chronic postoperative pain than open anterior mesh repair (Lichtenstein), with similar recurrence when performed by surgeons with sufficient experience [4]. Tissue repair (Shouldice) remains a valid option for carefully selected patients where expertise is available; in such circumstances, one-year outcomes can be comparable to those of Lichtenstein, TEP, and TAPP. While mesh is recommended for the majority, non-mesh repair may be offered after shared decision-making [4]. In our cohort, Shouldice repair was associated with less postoperative pain, fewer intraoperative and perioperative complications, and shorter operative times than laparoscopic TAPP.

Strengths and limitations

Our study has several strengths that enhance its credibility. The study was conducted prospectively in a comparative manner, with patients allocated to either Shouldice or laparoscopic TAPP repair based on a predefined sequence, which reduced selection bias and allowed for a fair comparison of outcomes. The sample size is relatively robust, boosting the statistical power of our findings and improving their applicability. Another strength is the extended 12-month follow-up, which enabled assessment of both early postoperative outcomes, such as pain, seroma, and wound complications, and longer-term issues, including inguinodynia, testicular atrophy, foreign body sensation, and recurrence. The use of standardized operative techniques across all cases further supports the reliability of the data.

Nonetheless, some limitations must be acknowledged. Although the allocation was structured, strict randomization with blinding was not feasible due to the surgical context; therefore, the study does not fully meet the CONSORT criteria for a randomized controlled trial. Cost and accessibility also represent important considerations: laparoscopic TAPP generally incurs higher expenses related to equipment and mesh, which may limit its feasibility in resource-constrained environments. In such instances, Shouldice repair retains an advantage as a tissue-based technique that is more accessible in lower-resource settings. While efforts were made to ensure patients were informed of both procedures and counseled about the financial implications before allocation, affordability may have influenced treatment acceptance and could represent an uncontrolled confounder. Additionally, while pain and functional outcomes were measured objectively, variables such as foreign body sensation relied on patient-reported assessments and remain inherently subjective. Finally, although 12 months of follow-up is substantial, certain chronic complications, especially late recurrences and mesh-related morbidities, may appear beyond this timeframe.

## Conclusions

Shouldice repair, a traditional tissue-based technique, remains a reliable option for repairing inguinal hernias. Operative time was shorter for the Shouldice repair compared with laparoscopic TAPP, while early complications such as pain were comparable between groups, and seroma was more frequent in the TAPP group. Chronic pain and foreign body sensation were also less common after the Shouldice repair. Our study contributes further evidence by providing prospective comparative data with 12-month follow-up, evaluating both early and long-term outcomes while accounting for unilateral versus bilateral repairs. Larger randomized controlled trials with longer follow-up are needed to confirm these findings, particularly regarding recurrence and late complications.
